# A Novel QTL Conferring *Fusarium* Crown Rot Resistance Located on Chromosome Arm 6HL in Barley

**DOI:** 10.3389/fpls.2019.01206

**Published:** 2019-10-15

**Authors:** Shang Gao, Zhi Zheng, Haiyan Hu, Haoran Shi, Jian Ma, Yaxi Liu, Yuming Wei, You-Liang Zheng, Meixue Zhou, Chunji Liu

**Affiliations:** ^1^Agriculture and Food, CSIRO, St Lucia, QLD, Australia; ^2^TIA, University of Tasmania, Prospect, TAS, Australia; ^3^Triticeae Research Institute, Sichuan Agricultural University, Wenjiang, Chengdu, China; ^4^School of Life Science and Technology, Henan Institute of Science and Technology, Xinxiang, China

**Keywords:** *Fusarium* crown rot (FCR), Barley (*Hordeum vulgare L.*), QTL, Breeding, RIL population

## Abstract

*Fusarium* crown rot (FCR), caused primarily by *Fusarium pseudograminearum*, is a devastating disease for cereal production in semi-arid regions worldwide. To identify and characterize loci conferring FCR resistance, we assessed a landrace AWCS799 which is among the best lines identified from a systematic screening of more than 1,000 genotypes. Genetic control of its resistance was investigated by generating and analyzing two populations of recombinant inbred lines with AWCS799 as the common parent. One of the populations was used for QTL detection and the other for validation. A novel QTL, located on the long arm of chromosome 6H (designated as *Qcrs.caf-6H*), was consistently detected in each of the four FCR severity tests conducted against the mapping population. The QTL explained up to 28.3% of the phenotypic variance, and its effect was confirmed in the validation population. Significant interaction between this resistance locus and either plant height or heading date was not detected, further facilitating its manipulation in breeding programs.

## Introduction

*Fusarium* crown rot (FCR) is a chronic disease caused by various *Fusarium* species in cereal crops. It is prevalent in arid and semi-arid cropping regions worldwide (Akinsanmi et al., 2004; Chakraborty et al., 2006). The disease has been known for causing significant yield loss in Australia (Murray and Brennan, 2009; Murray and Brennan, 2010) and the USA (Smiley et al., 2005b). FCR has also become a major issue in recent years for cereal production in China (Zhou et al., 2014; Li et al., 2016; Xu et al., 2017).

FCR pathogens are carried over in crop residues. They can survive for two seasons or longer in the field environments (Smiley et al., 2005a; Chakraborty et al., 2006), making the disease difficult to manage using practices such as crop rotation (Burgess, 2014). As recognized several decades ago, growing resistant varieties has to be an integral component in effectively managing the disease (Purss, 1966; Wildermuth and Purss, 1971). Growing resistant varieties does not only reduce yield losses but could also reduce the loss of the following barley or other cereal crops by reducing the inoculum load. This is especially the case for barley as, compared with those of wheat, its plants accumulate much higher concentrations of *Fusarium* pathogens at every stage of crown rot infection (Liu et al., 2012a).

The availability of well-characterized genotypes with high levels of resistance would facilitate breeding varieties with enhanced resistance. To date, there are only three reported studies on identifying QTL conferring FCR resistance in barley. The first one was based on a study using an existing population developed for unrelated traits. The study detected a single locus on chromosome arm 3HL, which interacts strongly with plant height (PH) (Li et al., 2009). Effects of PH on FCR development were also detected based on near isogenic lines (Chen et al., 2014) and histological analyses (Bai and Liu, 2015). The second study detected a single locus on chromosome arm 4HL (designated as *Qcrs.cpi-4H*) from a wild barley genotype (*Hordeum spontaneum* L.) originated from Iran (Chen et al., 2013a). The third study detected two loci from a landrace originated from Japan. They located on 1HL (*Qcrs.cpi-1H*) and 3HL (*Qcrs.cpi-3H*), respectively (Chen et al., 2013b).

Gene pyramiding has shown to be effective in further improving FCR resistance in both barley (Chen et al., 2015) and wheat (Zheng et al., 2017). However, as discussed above, only three loci conferring FCR resistance have been reported in barley. The value of one of them is also questionable as it interacts strongly with PH (Li et al., 2009; Chen et al., 2014). With the aim of identifying additional loci with high levels of resistance to FCR, we conducted a QTL mapping study against AWCS799, which was a landrace originated from South Korea. This genotype was identified as one of the most resistant genotypes from a systematic screening of more than 1,000 genotypes representing diverse geographical origins and different plant types (Liu et al., 2012b). As previous studies have repeatedly shown that both PH (Li et al., 2009; Ma et al., 2010; Chen et al., 2013a; Zheng et al., 2014; Bai and Liu, 2015) and heading date (HD) (Liu et al., 2012b) may interact with FCR severity, we investigated possible interactions between QTL detected from this study with these characteristics. Results obtained from these analyses are reported in this publication.

## Materials and Methods

### Plant Materials

The genotype AWCS799 is the resistant source analyzed in this study. Two populations of recombinant inbred lines (RILs) between “AWCS799” and two cultivars, Fleet and Franklin, were developed and used in this study. They are:

1. Fleet/AWCS799 consisting of 124 F8 RILs.
2. Franklin/AWCS799 consisting of 121 F8 RILs.

Both populations were produced in glasshouses at the Queensland Bioscience Precinct in Brisbane, Australia. The first population was used for QTL mapping and the other for validating putative QTL identified from the mapping population.

### FCR Inoculation and Disease Assessments

Results from previous studies show that FCR resistance is not pathogen species-specific, and the same resistance locus can be detected by pathogen isolates belonging to different *Fusarium* species (Ma et al., 2010; Chen et al., 2013b). We therefore used a single *F. pseudograminearum* isolate in this study. The isolate (CS3096) was obtained from a wheat field in northern New South Wales, Australia, and maintained in the CSIRO collection (Akinsanmi et al., 2004). Inoculum preparation, inoculations, and FCR assessments were as described by Li et al. (2008). Briefly, inoculum was prepared using plates of one-half-strength potato dextrose agar. Inoculated plates were kept for 12 days at room temperature before the mycelia were scraped and discarded. The plates were then incubated for a further 7 to 12 days under a combination of cool white and black fluorescent lights with 12-h photoperiod. The spores were then harvested, and the concentration of spore suspension was adjusted to 1 × 10^6^ spores/ml. The spore suspensions were stored in -20°C freezer, and Tween 20 was added (0.1% v/v) to the spore suspension prior to use.

Seeds were germinated in Petri dishes on three layers of filter paper saturated with water. Seedlings of 3 days old were immersed in the spore suspension for 1 min, and two seedlings were planted into a 3-cm^2^ punnet (Rite Grow Kwik Pots, Garden City Plastics, Australia) containing sterilized University of California mix C (50% sand and 50% peat v/v). The punnets were arranged in a randomized block design and placed in a controlled environment facility (CEF). Settings for the CEF were as follows: 25°C/18°C ( ± 1°C) day/night temperature and 65%/80% ( ± 5%) day/night relative humidity, and a 14-h photoperiod with 500 µmol m^−2^s^−1^ photon flux density at the level of the plant canopy. To promote FCR development, water stress was applied during plant growth. Inoculated seedlings were watered only when wilt symptoms appeared.

For QTL mapping, four independent FCR severity tests were carried out against the mapping population (designated as FCR01 to FCR04, respectively). Three independent tests were conducted on the validation population (designated as FCRV01, FCRV02, FCRV03, respectively). Fourteen seedlings for each of the RILs and both parental genotypes were used for each of the tests. *Fusarium* crown rot severity was assessed 4 weeks after inoculation, using a 0-point (no visible symptom) to 5-point (whole plant severely to completely necrotic) scale as described by Li et al. (2008). Mean of scores for each line was used as disease index (DI) in QTL analysis.

### Evaluation for PH and HD

To assess the possible effects of PH and HD on FCR resistance, a trial consisting of three replicates was conducted on the mapping population (Fleet/AWCS799) with randomized block design at the CSIRO Research Station at Gatton, Queensland (27°34′S, 152°20′E). For each replicate, 20 seeds for each of the RILF8 lines were sown in a single 1.5-m row with a 25-cm row spacing. Plant height was measured at maturity as the height from the soil surface to the tip of the spike (awns excluded). Six measurements were taken from the six tallest tillers in each row, and the average of each line was used for statistical analyses. Heading date was recorded on the day when 50% of all plants were at Zadock’s stage Z55 (Tottman et al., 1979).

### Molecular Marker Analysis

Genotypes for the two parents and 94 RILs from the population Fleet/AWCS799 were generated by the Department of Economic Development, Jobs, Transport and Resources, Victoria, Australia, according to a tGBS (an optimized approach for genotyping-by-sequencing) pipeline (Ott et al., 2017). Simple sequence repeats (SSR) markers were then developed for putative QTL regions and used to genotype the whole mapping population. SSR finder (https://github.com/GouXiangJian/SSR_finder) was used to screen the variants within QTL regions between the pseudomolecule of “Morex” (Mascher et al., 2017) and an assembly of a wild barley (*H. spontaneum*) genotype *AWCS276* (Liu et al., 2019). Primers were designed using Primer-BLAST (Ye et al., 2012).

PCRs for the amplification of the SSR markers were carried out in a total volume of 12 μl containing 25 ng genomic DNA, 0.2 μM of forward and reverse primer, 3 mM MgCL_2_, 0.2 mM dNTPs, and 0.5 U *Taq* DNA polymerase. During PCR, marker products were labelled with α-[^33^P]dCTP (3,000 ci/mmol). Polymerase chain reaction reactions were run on a Gene Amp PCR system 2700 thermocycler (PE Applied Biosystems, Foster City, CA, USA) programmed with the cycling conditions: one cycle of 5 min at 94°C, 35 cycles of 30 s at 94°C, 30 s at 60°C, and 1 min at 72°C, with a final extension step of 5 min at 72°C. The amplified products were mixed with an equal volume of loading dye, denatured at 95°C for 5 min, and 4 μl samples was run on a denaturing 5% polyacrylamide (20:1) gel at 110 W for 2 h. The gels were subsequently dried using a gel dryer for 30 min at 80°C and exposed to Kodak X-Omat X-ray films for 2 days.

### Data Analysis and QTL Mapping

To generate the best linear unbiased prediction (BLUP) for DI values from the four FCR severity tests, R package “lme4” (Bates et al., 2007) was used with the following mixed-effect model: *Y* *_ij_* = μ + *r* *_i_* + *g* *_j_* + *w* *_ij_*, where *Y* *_ij_* = DI value on the *j*th genotype in the *i*th test; μ = general mean; *r* *_i_* = effect due to *i*th test; *g* *_j_* = effect due to the *j*th genotype; *w* *_ij_* = error or genotype by test interaction, where genotype was treated as a random effect and that of test as fixed. The graph for the frequency distribution of DI values and Pearson correlation coefficient between all phenotypic data was generated with Microsoft Office Excel 2016 and R package “psych” (Revelle, 2017), respectively. Student *t* test was performed to evaluate the difference in DI values between lines with or without the resistant allele in the populations using Microsoft Office Excel 2016.

MSTmap Online (Wu et al., 2008) was used to build linkage maps by chromosomes with the following parameters: Grouping logarithm of the odds ratio (LOD) Criteria: single LG; Population type: RIL8; No mapping missing threshold: 10%; No mapping distance threshold: 15 centiMorgan (cM); No mapping size threshold: 2; Try to detect genotyping errors: Yes; Genetic mapping function: Kosambi. The diagrams of linkage maps were generated with MapChart (Voorrips, 2002). IciMapping 4.1 was used for QTL analysis with the “Biparental Population” (BIP) module (Meng et al., 2015). Inclusive composite interval mapping was applied to identify QTL with a mapping step of 1 cM (PIN = 0.015). For each FCR severity test, a 1,000-permutation test was performed to decide the LOD threshold corresponding to a genome-wide type I error less than 5% (*P* < 0.05). QTL were named according to the International Rules of Genetic Nomenclature (http://wheat.pw.usda.gov/ggpages/wgc/98/Intro.htm).

## Results

### Characterization of FCR Resistance in the Mapping Population of Fleet/AWCS799

**Figure 1:**
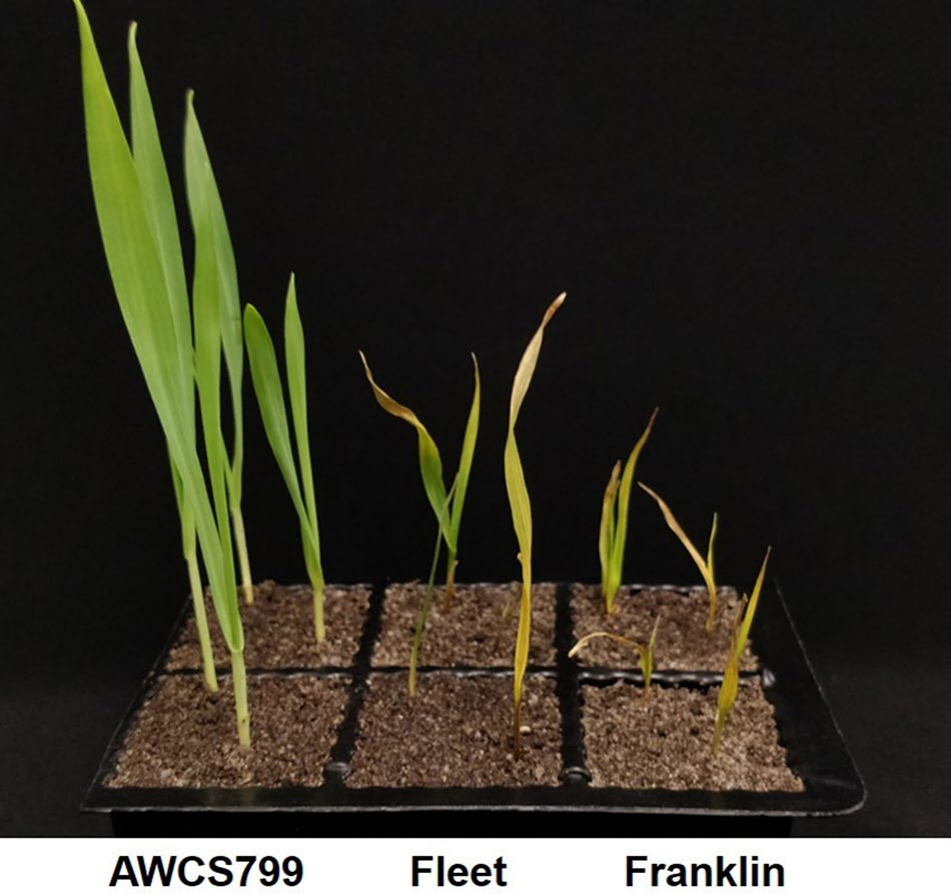
Difference in resistance to *Fusarium* crown rot (FCR) infection between the resistant genotype AWCS799 and the two commercial cultivars (Fleet and Franklin) used as parents in this study.

FCR severity of the resistant genotype AWCS799 was significantly lower than the two susceptible commercial cultivars (**Figure 1**). In the four FCR severity tests conducted against the mapping population and its two parents, the DI value of AWCS799 was 50.0% lower than that of Fleet on average, and transgressive segregation was detected in each of the tests (**Table 1**). DI values for all four tests and BLUP presented significantly positive correlation between each other (**Table 2**). The frequency distribution of DI value for FCR01 skewed towards better resistance. Disease index values for the other three tests and BLUP showed more normal distributions (**Figure S1**).

**Table 1:** Disease index of FCR severity in the population of Fleet/AWCS799.

Test	Parent		Population			
	Fleet	AWCS799	Min	Max	Mean	SD
FCR01	3.4	1.3	0.9	4.2	2.2	0.9
FCR02	3.4	1.5	1.1	4.7	2.9	0.8
FCR03	3.6	2.4	1.1	4.6	2.7	0.8
FCR04	4.2	2.1	1.4	4.4	3.0	0.7
BLUP	3.4	1.6	1.2	4.1	2.5	0.6

SD, standard deviation.

**Table 2:** Correlation coefficients between FCR severity, plant height, and heading date in the Fleet/AWCS799 population.

	FCR01	FCR02	FCR03	FCR04	BLUP	PH	HD
FCR01	1.00						
FCR02	0.23**	1.00					
FCR03	0.88*	0.29**	1.00				
FCR04	0.33**	0.25**	0.36**	1.00			
BLUP	0.79**	0.56*	0.83**	0.66**	1.00		
PH	−0.11	−0.10	−0.06	−0.10	−0.13	1.00	
HD	−0.10	−0.11	0.03	0.06	−0.08	0.35**	1.00

*Significant at P < 0.05; **significant at P < 0.01.

HD, heading date; PH, plant height.

### Linkage Maps Constructed and Synteny for Marker Locations in the Genome Assembly of “Morex”

Of the GBS markers mapped, 4,870 codominant markers were polymorphic between Fleet and AWCS799. These markers fell into 740 clusters and markers within each of the clusters cosegregated. As cosegregating markers contain the same information when used for mapping, a single marker with the least missing values was selected from each of the clusters and used for linkage map construction.

The markers were grouped into seven linkage groups, and they spanned a total of 1964.7 cM with an average distance of 2.3 cM between loci (**Table S1**; for details of linkage map, see **Table S2**). As all the markers generated had known physical positions, we aligned the linkage maps with the reference genome of barley based on the cultivar “Morex” (Mascher et al., 2017). This analysis found that, as expected, the genetic and physical maps were highly consistent for majority of the markers. However, there are a few exceptions around the pericentromeric regions (**Figure S2**).

### Detection and Validation of QTL for FCR Resistance

**Figure 2:**
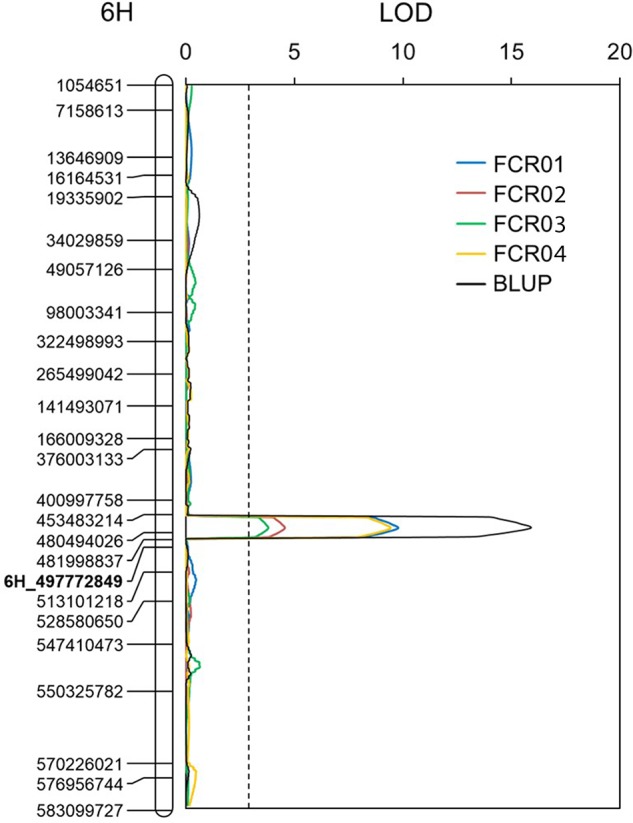
QTL conferring FCR resistance detected on the long arm of chromosome 6H in the population of Fleet/AWCS799. Physical position for each of the markers is shown to the left of the linkage map, and distances in centiMorgan (cM) are shown to the right. The vertical dotted lines indicate the average significance threshold (LOD = 2.7) based on a test of 1,000 permutations. The SSR marker used in validating the QTL is in bold.

Putative QTLs were detected on chromosomes 1H, 2H, 3H, 4H, 5H, and 6H. Only the one located on 6H was detected in each of the four tests conducted. The resistant allele of this QTL originated from AWCS799. We designated this QTL as *Qcrs.caf-6H*, where “crs” stands for “crown rot severity,” and “caf” represents “CSIRO Agriculture and Food.” Qcrs.*caf-6H* was identified in all four tests and BLUP, and it explained up to 28.3% of the phenotypic variance (**Figure 2** and **Table 3**). *Qcrs.caf-6H* was delineated into a 7.0-cM interval flanked by markers 6H_453483214 and 6H_481998837 with 6H_497772849 (**Table S3**) as the most closely linked SSR marker.

**Table 3:** Results of QTL analysis for FCR severity, plant height, and heading date identified in the population of Fleet/AWCS799.

Tests	QTL	Interval	Flanking markers	LOD	PVE (%)^a^	Origin
BLUP	*Qcrs.caf-6H*	118.5–125.5	6H_453483214 & 6H_481998837	14.6	28.3	AWCS799
	*Qcrs.caf-4H.1*	195.5–197.5	4H_613436155 & 4H_615703492	3.1	4.5	Fleet
	*Qcrs.caf-4H.2*	208.5–210.5	4H_630512237 & 4H_633453171	7.0	11.4	AWCS799
	*Qcrs.caf-2H*	194.5–197.5	2H_638031213 & 2H_639632684	3.0	4.4	AWCS799
	*Qcrs.caf-1H.1*	170.5–239.5	1H_488323557 & 1H_540988817	3.3	4.8	AWCS799
FCR01	*Qcrs.caf-6H*	118.5–125.5	6H_453483214 & 6H_481998837	9.8	12.7	AWCS799
	*Qcrs.caf-5H.1*	135.5–141.5	5H_410496043 & 5H_451983882	5.1	5.8	AWCS799
	*Qcrs.caf-5H.2*	144.5–146.5	5H_450803506 & 5H_459449531	10.3	13.5	Fleet
	*Qcrs.caf-4H.3*	47.5–51.5	4H_28747562 & 4H_32616055	3.4	3.7	AWCS799
	*Qcrs.caf-1H.1*	202.5–211.5	1H_520276315 & 1H_524761112	5.2	6.2	AWCS799
FCR02	*Qcrs.caf-6H*	118.5–125.5	6H_453483214 & 6H_481998837	4.6	15.9	AWCS799
	*Qcrs.caf-5H*	31.5–34.5	3H_26301091 & 3H_27911717	6.0	20.6	Fleet
FCR03	*Qcrs.caf-6H*	118.5–125.5	6H_453483214 & 6H_481998837	3.8	6.4	AWCS799
	*Qcrs.caf-1H.2*	81.5–86.5	1H_300306311 & 1H_134967717	4.7	8.0	Fleet
	*Qcrs.caf-1H.3*	95.5–98.5	1H_377585283 & 1H_358493268	8.2	15.1	AWCS799
FCR04	*Qcrs.caf-6H*	118.5–125.5	6H_453483214 & 6H_481998837	9.6	12.0	AWCS799
	*Qcrs.caf-4H.4*	92.5–95.5	4H_345503178 & 4H_362989846	8.9	10.8	AWCS799
	*Qcrs.caf-4H.1*	195.5–197.5	4H_613436155 & 4H_615703492	5.7	6.5	Fleet
	*Qcrs.caf-4H.2*	208.5–210.5	4H_630512237 & 4H_633453171	12.2	16.7	AWCS799
	*Qcrs.caf-1H.1*	170.5–239.5	1H_488323557 & 1H_540988817	3.0	3.3	AWCS799
PH	*Qcrs.ph-7H*	118.2–124.7	7H_180473897 & 7H_408998941	7.4	27.8	AWCS799
	*Qcrs.ph-6H*	160.5–163.3	6H_555603298 & 6H_552874297	3.7	11.7	Fleet
HD	*Qcrs.hd-5H*	248.4–252.4	5H_594490721 & 5H_599429072	14.4	48.9	AWCS799

a Percentage of phenotypic variance explained.

HD, heading date; PH, plant height.

Three QTLs were identified on chromosome 1H. Of them, only *Qcrs.caf-1H.1* was detected in more than one test, and it was located in a similar genomic interval with another FCR QTL reported in a previous study (Chen et al., 2013b). Four QTLs were detected on chromosome 4H. Of them, *Qcrs.caf-4H.1* and *Qcrs.caf-4H.2* were identified in both FCR04 and BLUP. *Qcrs.caf-4H.2* was mapped in a similar location with a locus reported by Chen et al. (2013a). The other two 4H QTLs and loci on chromosome 2H (*Qcrs.caf-2H*), 3H (*Qcrs.caf-3H*), and 5H (*Qcrs.caf-5H.1 and Qcrs.caf-5H.2*) were identified in only one of the tests. As none of these loci were consistently detected, they were not further investigated in this study.

Possible effects of *Qcrs.caf-6H* were further assessed in the validation population of Franklin/AWCS799. The most closely linked SSR marker with *Qcrs.caf-6H* from the mapping population was used to identify individuals with either the resistant (*RR*) or susceptible (*rr*) allele in this population. Significant difference was detected for *Qcrs.caf-6H* between *RR* and *rr* group in each of the three tests (**Table 4**). The average DI value of the lines bearing the resistant allele was 22.9% lower than those with the susceptible allele.

**Table 4:** Disease indices of FCR severity of lines possess resistant (*RR*) and susceptible (*rr*) allele of *Qcrs.caf-6H* from the population of Franklin/AWCS799.

Tests^a^	*RR* b	*rr* b	Difference (%)^c^	*P* d
FCRV01	3.0	3.7	21.0	<0.01
FCRV02	2.2	2.7	18.7	<0.05
FCRV03	1.9	2.6	28.9	<0.05

a The three tests conducted were designated as FCRV01, FCRV02, and FCRV03, respectively.

b The numbers of RR and rr genotypes are 50 and 71.

c Differences were obtained by comparison between RR and rr genotypes.

d P values were generated with Student t test.

### Effect of PH and HD on FCR Resistance

**Figure 3:**
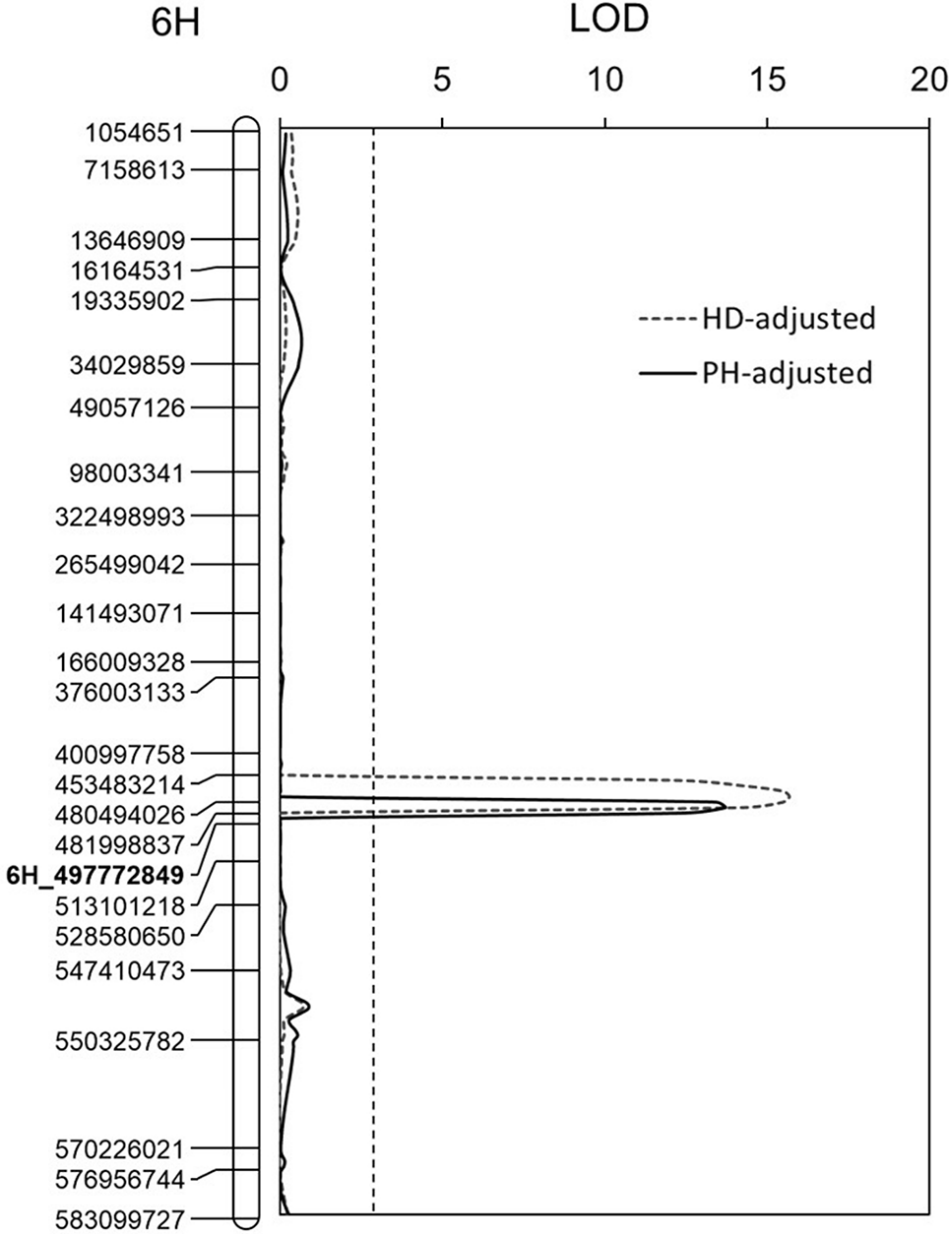
Effects of plant height (PH) and heading date (HD) on *Qcrs.caf-6H*. Physical position for each of the markers is shown to the left of the linkage map, and distances in cM are shown to the right. The LOD values were obtained from the BLUP and postadjustment by HD and PH. The vertical dotted lines indicate the average significance threshold (LOD = 2.7) based on a test of 1,000 permutations. The SSR marker used in validating the QTL is in bold.

A QTL controlling HD was identified on chromosome 5H. This QTL explained up to 48.9% of phenotypic variance with an LOD value of 14.4 (**Table 3**). Two QTL affecting PHs were detected and were located on chromosomes 6H and 7H, respectively. The QTL on chromosome 6H could explain up to 11.7% of the phenotypic variance with an LOD value of 3.7, and the other QTL on chromosome 7H could explain up to 27.8% of the phenotypic variance with an LOD value of 7.4 (**Table 3**). For quantifying possible effects of PH and HD on FCR severity, the BLUP of the four FCR severity tests was analyzed against PH and HD data using covariance analysis. The results showed that both PH and HD have little effect on *Qcrs.caf-6H* (**Figure 3**).

## Discussion

In the study reported here, we investigated the genetics of FCR resistance on a barley landrace originating from South Korea. A novel QTL located on chromosome arm 6HL was detected from each of the four tests. This QTL, designated as *Qcrs.cpi-6H*, explained up to 28.3% of the phenotypic variance in the mapping population and reduced FCR severity by 22.9% in the validation population on average. This is the first locus conferring FCR resistance identified on this chromosome in barley.

Pyramiding multiple loci into single genetic background have been proved to be effective in improving FCR resistance in barley and wheat (Chen et al., 2015; Zheng et al., 2017). However, the progress of such work in barley has been hampered by the shortage of effective loci. Of the only three loci reported in earlier studies, the value of the one located on chromosome arm 3HL is questionable as it had strong interaction with PH (Li et al., 2009; Liu et al., 2012a; Chen et al., 2013a). A histological analysis based on NILs for height also showed that *Fusarium pseudograminearum* hyphae were detected earlier and proliferated more rapidly during the time course of FCR development in the tall isolines (Bai and Liu, 2015). Some of the earlier studies showed that FCR severity can also be influenced by HD (Liu et al., 2012a). The new locus detected in this study does not colocate with loci controlling either PH or HD, and removing the effects of these characteristics from the mapping population has little influence on the magnitudes of the FCR locus detected in any of the tests. Thus, *Qph.caf-6H* is the third locus, which can be effectively exploited to further enhance FCR resistance by gene pyramiding in this crop species.

Loci conferring FCR resistance have been reported on 13 of the 21 wheat chromosomes (Liu and Ogbonnaya, 2015). One of the loci was located on wheat chromosome 6DL, and it was detected from a field trial (Martin et al., 2015). Compared with the 6DL locus, *Qph.caf-6H* seems to be more distally located on the chromosome arm 6HL in barley. However, as QTL mapping provides only limited resolution (Paterson et al., 1988), further studies are required to clarify the homologous relationship between these two loci.

As expected, orders for most of the markers in the linkage map constructed in this study aligned well with their physical positions in the barley genome. It is of interest to note that, without any exception, all the discrepancies involved markers and sequences located in the pericentromeric region (**Figure S2**). It is known that the pericentromeric regions of the barley chromosomes are characterized by low gene density, low recombination frequencies (Mascher et al., 2017), and high ratios of repetitive sequences (Wicker et al., 2017). Contributions, if any, from these characteristics to the discrepancies are not clear. However, it is not unreasonable to speculate that low recombination frequencies are likely to be less tolerable to incorrect marker scores or missing values occurred during high-throughput genotyping.

## Author’s Note

This study identified and validated a novel and large-effect QTL conferring Fusarium crown rot resistance on the long arm of chromosome 6HL in barley.

## Data Availability Statement

The raw data supporting the conclusions of this manuscript will be made available by the authors, without undue reservation, to any qualified researcher.

## Author Contributions

CL, MZ, YW, and Y-LZ conceived the research. SG, ZZ, HH, HS, JM, and YL conducted experiments and analyzed data under the supervision of CL and MZ. SG, ZZ, YL, and CL wrote the manuscript with contribution from all the authors.

## Funding

Work reported here was partially funded by the Grains Research and Development Corporation, Australia (Project CFF00010). SG is grateful to China Scholarship Council and the University of Tasmania for financial support during the tenure of his PhD studentship. HH thanks the Henan Institute of Science and Technology and China Scholarship Council for supporting her visit to CSIRO.

## Conflict of Interest

The authors declare that the research was conducted in the absence of any commercial or financial relationships that could be construed as a potential conflict of interest.
