# Supplementary material for: A Novel QTL Conferring Fusarium Crown Rot Resistance Located on Chromosome Arm 6HL in Barley
Source: Front Plant Sci. 2019 Oct 15;10:1206. doi: 10.3389/fpls.2019.01206 (PMC6803518; doi:10.3389/fpls.2019.01206)
Supplement: Supplementary file 1 [file DataSheet_1.pdf]

Table S1 Chromosome assignment and length of linkage groups, and marker density of the maps based on the population of Fleet/AWCS799

Chromosome	Number of markers	Length (cM)	Marker density (cM/Marker)
1H	84	256.3	3.1
2H	105	324.1	3.1
3H	165	356.7	2.2
4H	82	222.0	2.7
5H	134	343.8	2.6
6H	95	199.3	2.1
7H	76	262.5	3.5
Total	741	1964.7	2.7

Table S2 Table S2 A linkage map of barley based on the population of Fleet/AWCS799

Table S3 Primer sequences of the maker linked with *Qcrs.caf-6H*

Marker ID	Primer sequences	Product length (bp)	Motif / Number [a]	Tm (°C)
6H_SSR_497772849	5’-GCATTAGTTGTCATAGTAGGTAGCA-3’	242	C / 10	60
	5’-TTCAAGACCACGACCTTGGG-3’			

[a] The difference of motif number between two alleles.

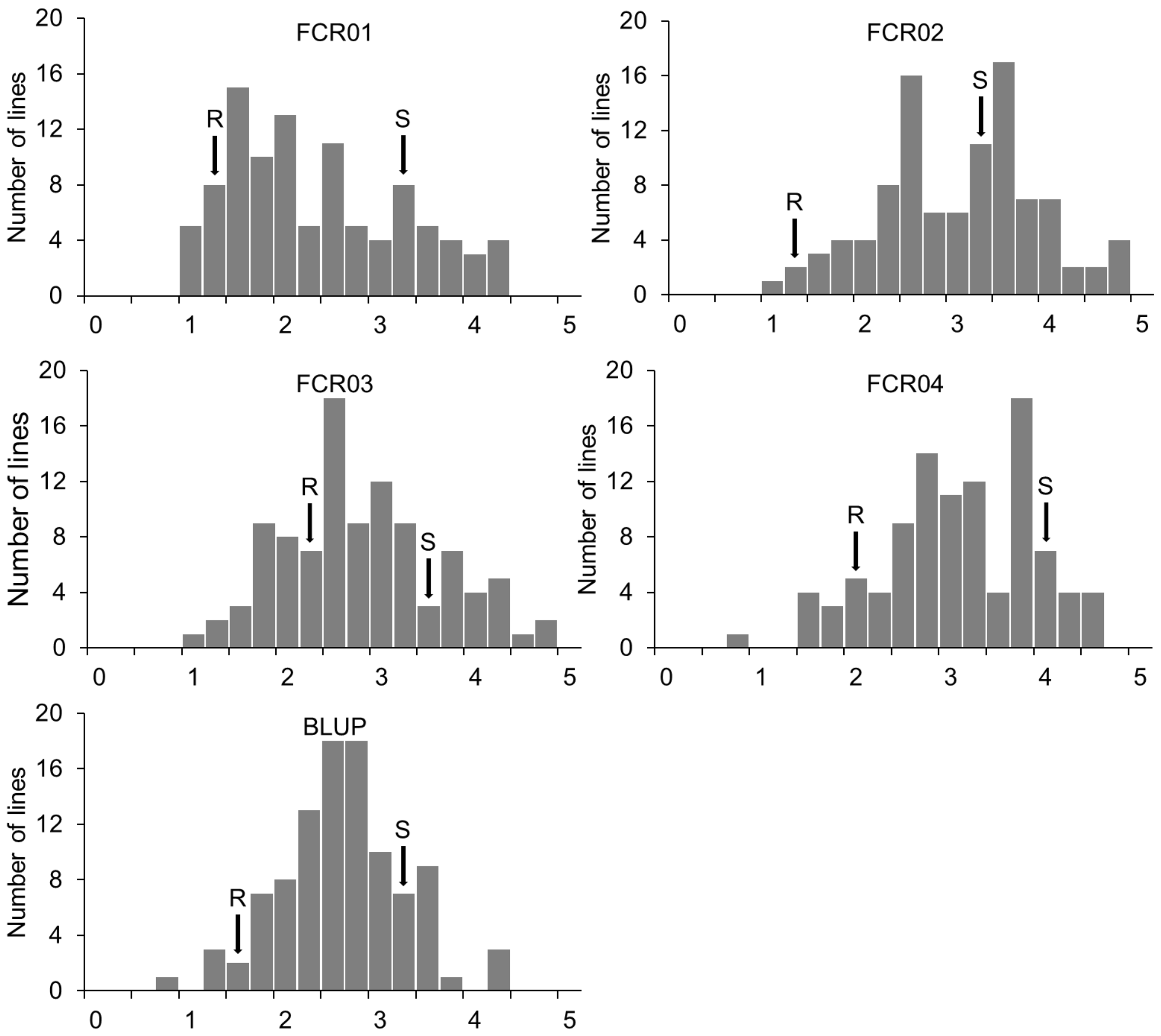

Fig. S1 Distribution of disease index (DI) values for the four FCR severity tests and the BLUP data from the mapping population Fleet/AWCS799. “S” stand for susceptible parent Fleet and “R” for resistant parent AWCS799.

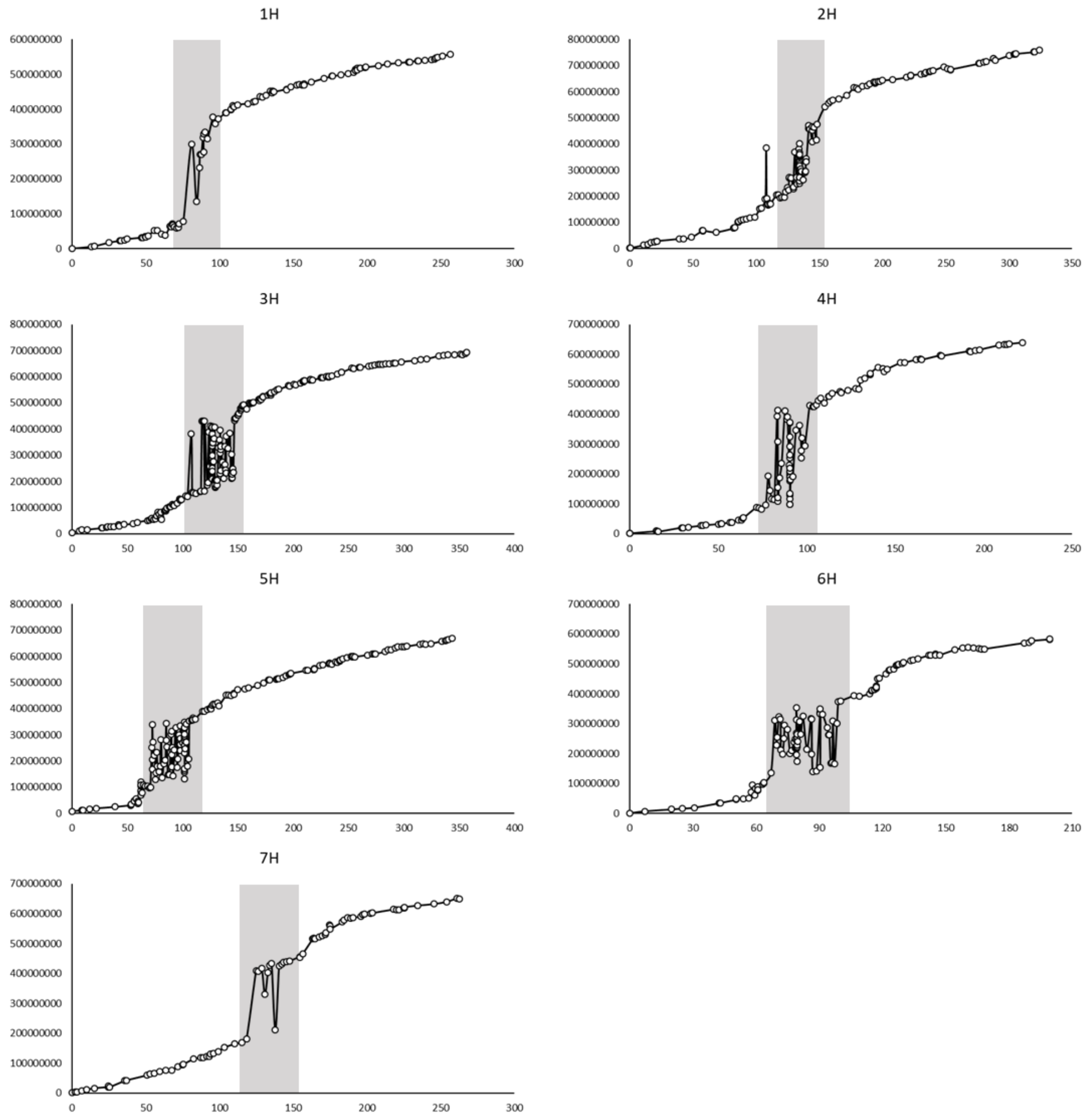

Fig. S2 Syntenic relationships for the mapped markers between the genetic and physical positions. Horizontal-axis represents genetic distance (cM) and vertical-axis represent physical distances (base pairs). Peri-centromeric regions were highlighted in gray shadow.
